# A pyoderma gangrenous-like cutaneous leishmaniasis in a Libyan woman with rheumatoid arthritis: a case report

**DOI:** 10.1186/s13104-018-3272-2

**Published:** 2018-03-01

**Authors:** Hamida Al-Dwibe, Ahmad Amro, Aisha Gashout, Ali El-Zurghany, Said El-zubi, Mohamed El-Hashme, Omar Hamarsheh, Mokhtar Maree

**Affiliations:** 1 Dermatology Department, Faculty of Medicine, University of Tripoli, Tripoli, Libya; 2 Faculty of Pharmacy, Al-Quds University, Jerusalem, Palestine; 3 Pathology Department, Faculty of Medical Technology, University of Tripoli, Tripoli, Libya; 4 Department of Biological Sciences, Faculty of Science & Technology, Al-Quds University, Jerusalem, Palestine

**Keywords:** *Leishmania*, Pyoderma gangrenous, Cutaneous leishmaniasis, Rheumatoid arthritis

## Abstract

**Background:**

Several case reports describe diseases presenting with skin ulcerations, which resemble pyoderma gangrenosum especially in immune-compromised patients, often proven on further workup, to have an infective or malignant etiology. However, treatment of pyoderma gangrenosum by systemic steroids or other immunosuppressive agents may worsen the condition.

**Case presentation:**

We report here, a 45 year-old Libyan woman with rheumatoid arthritis on low dose steroids with pyoderma gangrenosum-like skin lesions and positive pathergy. Slit–smear was positive for *Leishmania* amastigotes and histopathological examination confirmed the diagnosis of cutaneous leishmaniasis. The lesions healed completely by parenteral sodium stibogluconate (Pentostam) 600 mg daily.

**Conclusion:**

We report for the first time, a rare and unusual presentation of pyoderma gangrenosum like-cutaneous leishmaniasis in a patient with rheumatoid arthritis. Atypical cutaneous leishmaniasis should not be ruled out in the differential diagnosis of unresponsive skin diseases, with slit/smear and a skin biopsy is required.

**Electronic supplementary material:**

The online version of this article (10.1186/s13104-018-3272-2) contains supplementary material, which is available to authorized users.

## Background

Cutaneous leishmaniasis (CL) is a protozoan skin infection caused by various species of *Leishmania* parasites and transmitted by correspondent species of sand flies. CL is highly endemic in Libya especially after the armed conflict that outbreak in the country [1]. CL has a wide clinical spectrum, presenting in different clinical forms of the disease depending particularly on host immune response rather than on the parasite species [2, 3]. However, there are some clinical manifestations that are more prevalent in one host than in another [4, 5]. Unusual clinical variants of CL include mucocutaneous leishmaniasis (MCL), diffuse cutaneous leishmaniasis (DCL), disseminated cutaneous leishmaniasis (DCL), and leishmaniasis recidivans (LR). Rare forms of CL were reported with sporotrichoid, psoriasiform, and zosteriform [2, 6, 7]. In immune-compromised patients, skin ulcerations are often associated with infections or malignancy [8]. However, treatment of pyoderma gangrenous by systemic steroids or other immunosuppressive drugs proved to worse the condition. Herein, we report for the first time, a rare and unusual presentation of PG like CL in a patient with rheumatoid arthritis (RhA).

## Case presentation

A 46-year-old, women from Tawirgha, Libya was referred to the Leishmaniasis Clinic, Tripoli Central Hospital. She was admitted to the clinic with multiple papules, pustules, ulcerated nodules, and painful ulcerative lesions over upper and lower extremities for 2 years. The lesions started as painful papules and pustules, which gradually enlarged in size, and eventually ulcerated with pus discharge. Her worsened conditions, prompted her to seek advanced medical care. Two years later, the patient had been admitted twice to the hospital in her local village for the same problem. She was treated with topical and systemic antibiotics without any improvement, so she was referred to the Leishmaniasis Clinic in Tripoli Central Hospital. The patient had suffered from rheumatoid arthritis for 22 years, hypertension, and diabetes mellitus and has been prescribed a low dose of prednisolone (20 mg/days) for 20-years, beta-1 selective adrenergic antagonist, and an oral antihyperglycaemic medication.

No previous history of trauma or insect bites were reported by the patient. However, she had a history of CL in the buttocks, which was treated with intra-lesional injections of pentostam.

**Fig. 1:**
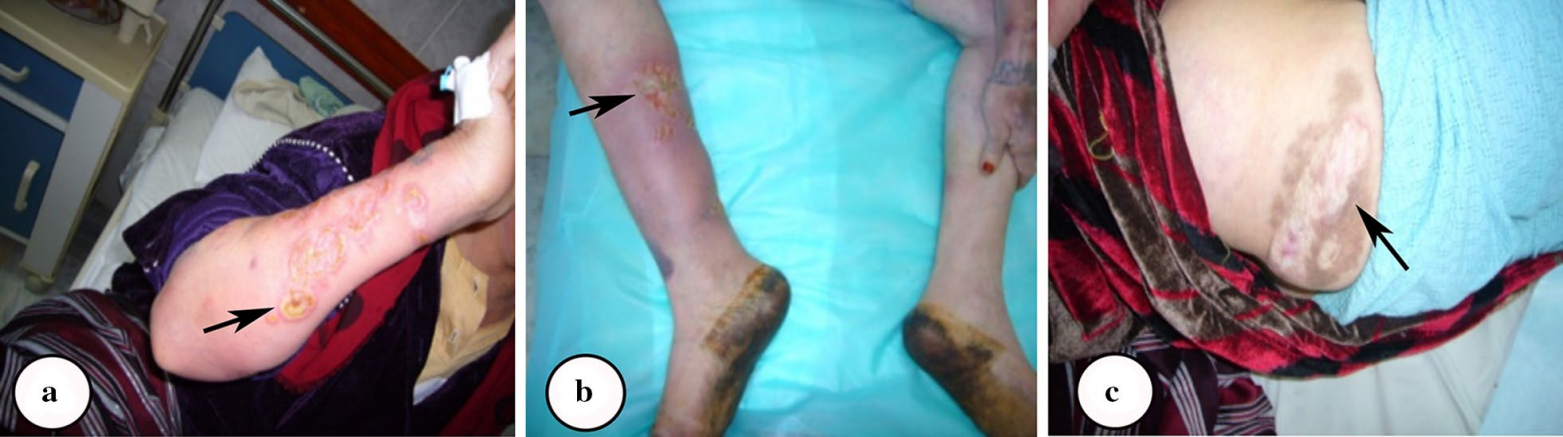
Deep ulcers on different regions of the patient extremities. **a** Multiple well defined erythematous ulcers with raised irregular indurated margins on one of the forearms. **b** Ulcers on flexural surfaces of the right leg associated with swollen inflamed surrounding skin. **c** A large hypo pigmented atrophic scar surrounded by a rim of hyperpigmentation over right buttock (healed CL lesions)

Physical examination revealed good general condition, a red puffy face with telangiectasia over both checks and mild lower limb edema. On cutaneous examination, the patient was found to have multiple erythematous papules, pustules, and ulcerated nodules as well as painful well defined erythematous ulcers of varying sizes with raised irregular indurated margins on both forearms (Fig. 1a) and multiple painful punched out ulcers on lower limbs (Fig. 1b). The base of ulcers contained yellowish exudate and necrotic yellowish slough (Fig. 1a, b). The ulcers on extensor and flexore surfaces on the right leg was associated with swelling of the leg with surrounding skin inflammation (Fig. 1b). In addition, large hypo pigmented atrophic scare surrounded by a rim of hyper-pigmentation over the right buttock (healed CL lesions), and pustules at site of injections were observed (pathergy) (Fig. 1c).

Skin examination revealed ulcers presenting as PG, or as vasculitic ulcers secondary to Rh.A or an unusual variant of CL. Routine hematological investigations revealed elevated white blood cell count (12.3 × 10^9^/l), hemoglobin level of 11.7 g/dl, platelet counts of 230 × 10^9^/l, ESR 15/h, CRP of 54 mg/l, FBS of 135 mg/dl and high levels of transaminases (GGT 528 U/l, GPT 80 U/l). Renal function tests, rheumatoid factor, and cortisol were at normal levels. Ultrasonography examination of the abdomen revealed enlarged liver with marked fatty changes. Serologic tests for HIV and viral hepatitis were negative. Chest radiograph, ECG, and Echo were normal.

**Fig. 2:**
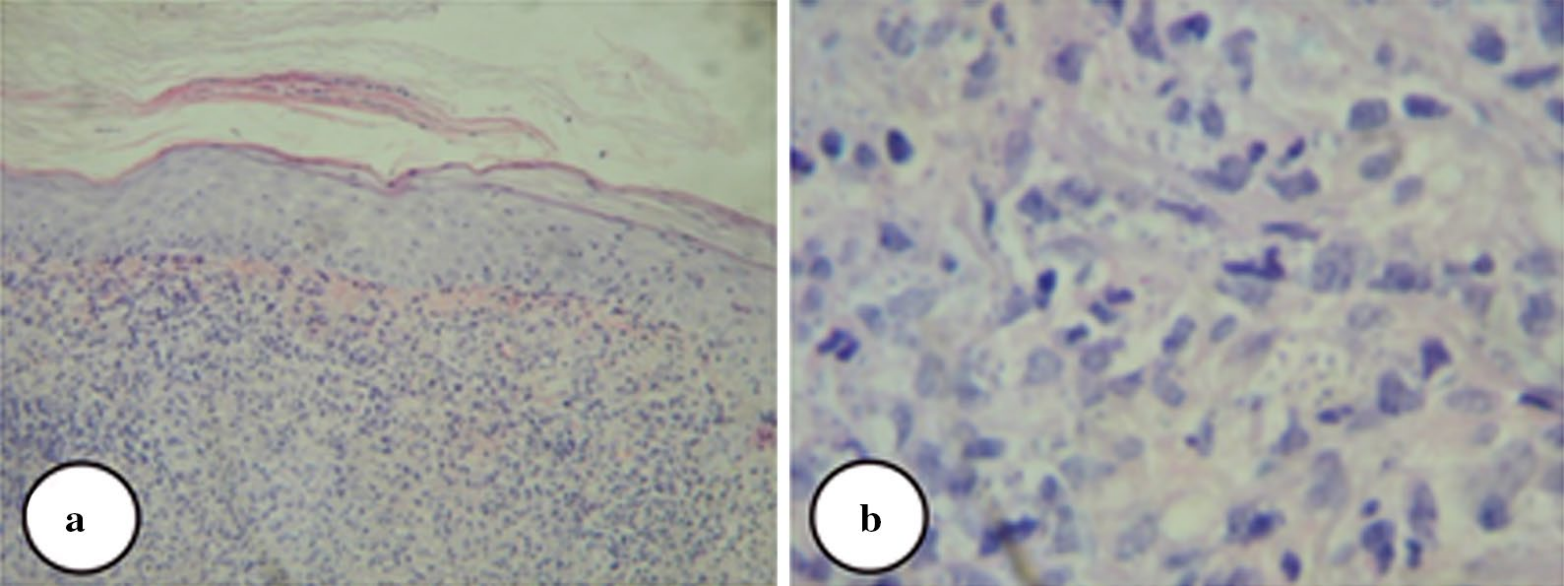
Histopathological analysis of skin biopsy. **a** The histopathological features of skin biopsy obtained from papulo–pustular lesions, **b** *Leishmania* parasites inside and outside macrophages

A skin biopsy obtained from the lesions revealed hyperkeratosis, focal parakeratosis, acanthosis, spongiosis, and exocytosis. Dermis showed heavy diffuse granulomatous infiltrate composed of epithelioid cells, and lymphocytes admixed with plasma cells, neutrophils, and mast cells with some extravasated RBCs (Fig. 2a). A perivascular chronic inflammatory cell infiltrate with extravasated RBCs, nuclear dust, thickening of endothelium and fibrinoid necrosis of few blood vessels and few *Leishmania* parasites was seen especially in biopsies obtained from leg ulcers. However, biopsies taken from the papulo–pustular lesions showed many *Leishmania* parasites inside and outside the macrophages (Fig. 2b). Molecular identification of the causative *Leishmania* spices was not done.

The patient was treated with systemic antibiotics, and sodium stibogluconate (Pentostam) (600 mg daily-I.M) was started after reduction of the transaminases enzymes by stopping all medications that have been used by the patient for long time. Complete healing of lesions was achieved after 15 days, except a small erythematous papule on the left forearm which was treated by cryotherapy. After 2 years follow up, no relapse was noted. The important milestones related to diagnosis, interventions and follow up of this case are listed in Additional file 1.

## Discussion and conclusions

Cutaneous leishmaniasis has been endemic in Northwestern regions of Libya for a long time. The incidence of this infection is rising since the year of 1971, and new foci have been reported recently [5]. Clinically, CL can resemble many skin diseases such as bacterial skin infections, fungal skin infections, mycobacterial infections, eczema, sarcoidosis, insect bites, and malignancies like basal and squamous cell carcinomas, and can be misdiagnosed as other diseases [9, 10]. When the ulcerating lesion of CL is located on the extremities especially legs, other diseases like pyoderma gangrenosum, a typical mycobacterial infection, venous, and arterial ulcers must be considered in the differential diagnosis [6]. In addition, venous, infective, and inflammatory leg ulcers such as vasculitis or pyoderma gangrenosum may develop in patients with rheumatoid arthritis. These ulcers are often painful, difficult to heal, and may last for years as described in this case.

The clinical diagnosis of CL was suspected since this patient came from an endemic area, having multiple lesions on exposed parts of body, not responding to treatment with antibiotics, had positive personal and family history of CL which are confirmed as risk factors for CL [11, 12] as well as confirmation of the case by a slit–skin smear and histopathological examination. The reason for this rare unusual clinical type of Pyoderma gangrenosum like CL is unknown and raises speculation and assumptions about the host parasite relationship and the capacity of *Leishmania* parasites to modulate the host immune response [13–16]. The infection by specific *Leishmania* strains, and an altered host immune response caused by systemic administration of steroid drugs may have a role for persistence of the lesions. However, patients on systemic corticosteroids are likely to be immune-compromised with increased susceptibility to infections, particularly with intra-cellular microorganisms, which may lead to the development of unusual types of leishmaniasis as explained by the blockade of cytokine expression by glucocorticoids released from T lymphocyte cells [17]. Pentavalent antimony compounds such as sodium stiboglyconate and meglumine antimoniate still remain the first choice for the treatment of CL [2–4, 6, 7, 18]. Sodium stiboglyconate 600 mg/day intramuscularly for 15 days was sufficient to completely heal the lesions in this case.

Atypical CL should not be ruled out in the differential diagnosis of unresponsive skin diseases, with slit/smear and a skin biopsy being required. Moreover, history of previous exposure to sand fly bites or previous *Leishmania* infection may help in the diagnosis. According to the author’s knowledge, this case represented the first reported case of CL presenting as pyoderma gangrenosum like lesions in Libya.

## Additional file

**Additional file 1.** Time line: Important milestones related to diagnosis, interventions and follow up of the case.

## Abbreviations

CL: cutaneous leishmaniasis
DCL: diffuse cutaneous leishmaniasis
LR: leishmaniasis recidivans
MCL: mucocutaneous leishmaniasis
PG: pyoderma gangrenosum
