# Supplementary material for: A pyoderma gangrenous-like cutaneous leishmaniasis in a Libyan woman with rheumatoid arthritis: a case report
Source: BMC Res Notes. 2018 Mar 1;11:158. doi: 10.1186/s13104-018-3272-2 (PMC5831575; doi:10.1186/s13104-018-3272-2)
Supplement: Supplementary file 1 — Additional file 1. Time line: Important milestones related to diagnosis, interventions and follow up of the case. [file 13104_2018_3272_MOESM1_ESM.docx]

| Dates | Relevant Past Medical History and Interventions | | |
| --- | --- | --- | --- |
| Since 1986 | The patient had Rh. Arthritis and treated on systemic steroid (20mg daily), Hypertension and treated with Atenolol (100mg/day), and  Diabetes mellitus on diet control. | | |
| Date | Summaries from Initial and Follow-up Visits | Diagnostic Testing  (including Dates) | Interventions |
| 26/2/2008  28/2/2008  28/2/2008  1/3/2008 | Dx-D-pyoderma gangrenosum, deep fungal infection, unusual cutaneous ulceration.  Uncontrolled sugar level in blood.  Patient complained of itchy lesions and development new skin lesions in perianal area.  Patient suffered from Chest pain radiating up to shoulders. | Microscopic examination of biopsies from lesion in the forearm.  Blood Sugar or Blood glucose tests FBS/RBS and HbA1C. 7(high)  Tissue scrapings from perianal area for potassium hydroxide (KOH) examination diagnosed fungal infection.  (1/3/2008) ECG test (normal),  tests for Cardiac enzymes (normal except LDH found high(308U/L)  (10/3/2008) repeated ECG test (normal) | Initially patient put on Insulin sliding scale, then on Metformin (500mg twice/day) only.  Micoter cream twice/day  Advised to take rest and use pain killers (Paracetamol) whenever needed. |
| 5/3/2008 | Patient complained abdominal pain and distension. | - Abdominal examination using ultrasound (revealed enlarged fatty liver (nonalcoholic steatosis hepatitis) and distended gall bladder - Viral screen for HIV/HCV/HBV (negative). | A Gastroenterologist advised to test for antimitochondrial Abs to rule out primary biliary cirrhosis Or drug induced hepatitis due to high LFT—especially—GGT  The patient reported about taking a mixture of traditional preparations mixed with honey, dramatic decrease of LFT was noticed when the patient stopped taking these traditional preparations. |
| 10/3/2008 |  | KOH examination was carried out from finger nails and was found positive for candida infection. | Micoter cream (Clotrimazole 1% Betamethasone 0.05%) was prescribed (twice daily) |
|  |  | Cortisol level was tested two times and found normal. |  |
|  |  | ESR test carried out several times —3, 10, 15, 28 mm/ 1hr |  |
| 15/3/2008 | Unresponsive treatment with antibiotics suspects of CL infection. | Microscopic examination of biopsies from skin lesions. | Sodium stiboglyconate (600mg/day) intramuscularly |
| 1/4/2008 | Lesions healed completely | Histopathological examination |  |
| 30/4/2008-1/4/2009 | Follow up | No relapses |  |
